# Preclinical Assessment of Mesenchymal-Stem-Cell-Based Therapies in Spinocerebellar Ataxia Type 3

**DOI:** 10.3390/biomedicines9121754

**Published:** 2021-11-24

**Authors:** Joana Sofia Correia, Andreia Neves-Carvalho, Bárbara Mendes-Pinheiro, Joel Pires, Fábio Gabriel Teixeira, Rui Lima, Susana Monteiro, Nuno André Silva, Carina Soares-Cunha, Sofia Cravino Serra, Sara Duarte-Silva, Andreia Teixeira-Castro, António José Salgado, Patrícia Maciel

**Affiliations:** 1 Life and Health Sciences Research Institute (ICVS), School of Medicine, University of Minho, 4710-057 Braga, Portugal or id8212@alunos.uminho.pt (J.S.C.); andreiacarvalho@med.uminho.pt (A.N.-C.); id7153@alunos.uminho.pt (B.M.-P.); jppdrg4@hotmail.com (J.P.); fabioteixeira@med.uminho.pt (F.G.T.); id6527@alunos.uminho.pt (R.L.); susanamonteiro@med.uminho.pt (S.M.); nunosilva@med.uminho.pt (N.A.S.); carinacunha@med.uminho.pt (C.S.-C.); sofiaserra@med.uminho.pt (S.C.S.); sarasilva@med.uminho.pt (S.D.-S.); accastro@med.uminho.pt (A.T.-C.); asalgado@med.uminho.pt (A.J.S.); 2 ICVS/3B’s—PT Government Associate Laboratory, 4710-057 Braga, Portugal

**Keywords:** human mesenchymal stem cells, secretome, neurodegeneration, spinocerebellar ataxia type 3, preclinical trial

## Abstract

The low regeneration potential of the central nervous system (CNS) represents a challenge for the development of new therapeutic strategies for neurodegenerative diseases, including spinocerebellar ataxias. Spinocerebellar ataxia type 3 (SCA3)—or Machado–Joseph disease (MJD)—is the most common dominant ataxia, being mainly characterized by motor deficits; however, SCA3/MJD has a complex and heterogeneous pathophysiology, involving many CNS brain regions, contributing to the lack of effective therapies. Mesenchymal stem cells (MSCs) have been proposed as a potential therapeutic tool for CNS disorders. Beyond their differentiation potential, MSCs secrete a broad range of neuroregulatory factors that can promote relevant neuroprotective and immunomodulatory actions in different pathophysiological contexts. The objective of this work was to study the effects of (1) human MSC transplantation and (2) human MSC secretome (CM) administration on disease progression in vivo, using the CMVMJD135 mouse model of SCA3/MJD. Our results showed that a single CM administration was more beneficial than MSC transplantation—particularly in the cerebellum and basal ganglia—while no motor improvement was observed when these cell-based therapeutic approaches were applied in the spinal cord. However, the effects observed were mild and transient, suggesting that continuous or repeated administration would be needed, which should be further tested.

## 1. Introduction

The burden of neurodegenerative diseases is growing with the aging of the population. Degeneration of neurons in the cerebellum, brainstem, and spinocerebellar tracts causes ataxia (reviewed in [1]). To date, many different types of inherited ataxia have been defined [2], including spinocerebellar ataxias (SCAs). Among SCAs, autosomal-dominant inherited polyglutamine (polyQ) diseases are the most frequent, of which SCA3/Machado–Joseph disease (MJD) is the most common. PolyQ diseases result from gene mutations that cause abnormally long polyglutamine tracts in the corresponding disease proteins, which act in a dominant toxic manner, and are associated with protein misfolding [3]. SCA3/MJD is a progressive disorder with a highly variable clinical presentation, which includes ataxia, ophthalmoplegia, amyotrophy, dystonia, and/or spasticity, frequently leading to premature death [4]. Postmortem analysis of patient brains reveals degeneration of the deep nuclei of the cerebellum, pontine and subthalamic nuclei, substantia nigra, and spinocerebellar tracts [5,6]. Despite the efforts of different research teams towards testing different molecular strategies to treat SCA3/MJD—such as chaperone induction [7], activation of autophagy [7,8,9,10], caloric restriction [11], modulation of intracellular calcium homeostasis [12], gene silencing [13,14,15,16], antioxidant treatment [17], HDAC inhibition [18,19], increasing serotonergic signaling [20], and neural stem cell transplantation [21]—an effective treatment to halt the progression of this fatal disease is not yet available (reviewed in [22]). Recent advances in stem cell technology aim at fulfilling this and other unmet medical needs (reviewed in [23,24]); it is crucial, however, to address important issues such as safety, delivery routes, dosage, duration, and efficacy in order to successfully translate the application of cell-based therapies for neurodegenerative disorders. Treatment strategies typically focus on cellular replacement, by grafting the cells into affected areas, expecting that they will differentiate into the specific neuronal subtypes lost in disease, integrate synapses, and regenerate a neuronal network similar to the one lost. Simultaneously, this cell transplantation could also provide environmental enrichment to support the affected neurons by producing neurotrophic factors, scavenging toxic factors, and/or creating auxiliary neural networks (reviewed in [23]). While very interesting work is being developed using neural progenitor cells [21], mesenchymal stem cells (MSCs) have been proposed as promising therapeutic tools for neurodegenerative disorders, allowing for increased reproducibility and translation potential, because these cells (1) are easy to isolate, (2) are able to greatly expand while retaining multipotent potential, (3) possess the ability to migrate toward neural lesions upon transplantation [25], and (4) can be safely used for autologous transplantation, exhibiting no toxicity or tumorigenicity when transplanted into rodents or human patients, opening a door for their use in human clinical trials [25,26,27,28,29,30,31,32]. In addition, MSCs safeguard themselves from the immune system [33,34], which potentially enables allogeneic transplantation [35]. Several possible routes for the transfer of factors from MSCs to neurons have been suggested (reviewed in [34]). Importantly, injected MSCs are likely attracted by chemokines secreted by degenerating neurons [28,36], making contact with them, and may thus directly provide neurotrophic factors to these cells, rescuing them from degeneration. Neurons might receive these factors directly through the interstitial space, via a special machinery (such as exosomes, tunneling nanotubules, or gap junctions), and/or via cell fusion. In addition to their differentiation potential, it is now well accepted that, in addition to their ability to repopulate neurodegenerated areas, the beneficial actions of MSCs can also result from their bystander capacities, such as the persistent paracrine secretion of a variety of bioactive factors—their secretome—in response to the tissue context, which offers a broad clinical potential [37]. In fact, it seems unlikely that the benefits of MSC transplantation mainly depend on their replacement potential; first, the number of viable and differentiated cells found at the end of the preclinical trials performed thus far has been very small and, second, functional cell replacement requires the establishment of physiologically functional connections via the integration of new MSC neurons into neuronal networks, which is difficult to accomplish [38,39]. Multiple recent reports have shown improvement in various models of neurodegenerative diseases following MSC transplantation, including spinal cord lesions [40,41], Parkinson’s disease [42,43,44], and various models of SCAs [39,45,46]. Beneficial effects include the mitigation of central and peripheral neurodegeneration, as well as the improvement of motor coordination. These previous findings make it very likely that similar treatments will be effective against SCA3/MJD—a hypothesis that we specifically tested in a mouse model that recapitulates key hallmarks of the human disease [7]. The CMVMJD135 mouse model has been extensively characterized by us and by other laboratories [47], constituting an excellent model to study the pathogenic mechanisms of SCA3, as well as for drug testing, as it resembles the human disease closely. This transgenic mouse line expresses human ataxin-3 with approximately 135 glutamines under the control of the CMV promoter, leading to a ubiquitous expression of ataxin-3; the expression levels of the mutant protein are close to those of the murine endogenous protein. Thus, this model adheres to the construct validity principle. The phenotype of the CMVMJD135 mice mimics the human condition well. The disease symptoms appear gradually, and progress slowly as the animals age. The first observed symptom is loss of muscular strength, starting as early as 6 weeks of age. Motor, balance, and gait deficits follow, appearing between 10 and 14 weeks of age. Loss of weight gain and reduced exploratory behavior are detectable from week 16 onwards. From week 18 it is also possible to observe a clear kyphosis. Later in life, these mice show abnormal neurological reflexes, including limb clasping and grasping, as well as tremors. Additionally, these mice have a reduced lifespan. Neuropathological findings present in the CMVMJD135 mice include (1) cellular loss in the pontine and dentate nuclei, the substantia nigra, and the spinal cord; (2) a decrease in calbindin-positive Purkinje cells, as well as reduced thickness of the molecular layer of the cerebellum; (3) loss of cholinergic neurons in the facial nuclei and spinal cord; (4) a reduction in the number of TH-positive neurons, and astrogliosis in the substantia nigra; (5) gross brain atrophy (late in life); (6) ataxin-3 intranuclear inclusions in several brain regions; and (7) reduced glucose metabolism. Importantly, although the onset of the symptoms occurs very early in this model (at 6 weeks of age), the onset of the neuropathological features occurs later in life—around 30 weeks of age. In summary, the CMVMJD135 model complies with the face validity criterium.

Clinical trials involving MSC transplantation in SCA patients (http://www.clinicaltrials.gov, NCT01360164, NCT01649687; accessed on 25 July 2021) have already been performed, with researchers reporting that the motor amelioration and delay in disease progression were transient, with some patients regressing a few months after treatment to a symptomatic severity similar to that prior to treatment [26,35,48,49]. However, these studies also point to the need to perform larger longitudinal, randomized, double-blinded, placebo-controlled trials to safely draw conclusions about the efficacy of this treatment. Meanwhile, these reports also prompt the search for less invasive treatments. Furthermore, there are some concerns regarding the standardization and storage of MSCs and their secretome [50,51]. One major obstacle that hampers the interpretation of preclinical/clinical research data obtained with MSC therapies stems from the fact that the effects of MSCs and MSC-based products vary because of their intrinsically heterogeneous cell composition, and may also change depending on their cell source and the protocols applied for cell isolation and expansion [52]. For this reason, it is crucial to use consistent cell sources and experimental protocols. In this work, we aimed at comparing the therapeutic efficacy of MSC transplantation in different CNS regions known to be relevant for SCA3/MJD, using approaches that proved effective in models of other neurodegenerative diseases, and comparing the effects of cell transplantation versus secretome administration, so as to contribute to refining the strategies for human translation.

## 2. Materials and Methods

### 2.1. Ethics Statement

All procedures were carried out in compliance with European regulations (European Union Directive 86/609/EEC). Animal facilities and the people directly involved in animal experiments (J.S.C., A.N.-C., B.M.-P., F.G.T., R.L., S.M., N.A.S., C.S.-C., S.D.-S.), as well as the principal investigators (A.J.S. and P.M.), were certified by the Portuguese regulatory authority Direção Geral de Alimentação e Veterinária. The ARRIVE guidelines were followed while reporting the results obtained in this study.

### 2.2. hMSC Culture, Secretome Collection and Concentration

Human MSCs (hMSCs) derived from bone marrow (Lonza, Switzerland) were cultured, and a conditioned medium (CM) was prepared, as previously described [53,54]. Cells were thawed and plated in T-75 culture flasks with alpha-MEM growth medium (21090-022, Thermo Fisher Scientific, Waltham, MA, USA) supplemented with fetal bovine serum (FBS; S0115, Biochrom, Berlin, Germany). When the cells reached 80% confluence, they were enzymatically dissociated using 0.05% trypsin-EDTA (25300-054, Thermo Fisher Scientific, Waltham, MA, USA) for 5 min at 37 °C. After that, cells were centrifuged at 1200 rpm (4 °C; Megafuge 1.0 R, Heraeus, Hanau, Germany) for 5 min. The supernatant was removed and the pellets were resuspended in fresh growth medium, from which a small volume of cells was diluted in trypan blue (T6146, Sigma, St. Louis, MO, USA) to perform viable cell counts. Finally, the cells were plated in culture flasks at a density of 4000 cells/cm^2^. At passage 5 (P5), after 72 h of growth, the alpha-MEM medium was removed and the cells were washed three times in phosphate-buffered saline (PBS) without Ca^2+^/Mg^2+^ (10010-015, Thermo Fisher Scientific, Waltham, MA, USA), and with Neurobasal-A medium (10888-022, Thermo Fisher Scientific, Waltham, MA, USA) supplemented with 1% kanamycin (15160-047, Life Technologies, Carlsbad, CA, USA). Cells were incubated in Neurobasal-A medium for 24 h, for secretome conditioning and collection. After 24 h, this medium, containing the factors secreted by hMSCs—the hMSC secretome (herein referred to as conditioned medium (CM))—was collected and centrifuged at 1200 rpm (Megafuge 1.0 R, Heraeus, Hanau, Germany) for 10 min to remove any cell debris. Afterwards, the CM was concentrated (100×) via centrifugation using a 5 kDa cutoff concentrator (Vivaspin 20, GE Healthcare, Chicago, IL, USA), and frozen at −80 °C until used for surgical procedures, as previously described [53]. Additionally, we performed total protein quantification of the CM (injected locally in the animals 100× concentrated) via the Bradford assay (based on the microassay protocol from Bio-Rad; #5000205, Bio-Rad, Hercules, CA, USA).

### 2.3. Animal Housing Conditions

Male CMVMJD135 (background C57BL/6) mice and wild-type littermates were used in this study. Animals were housed at weaning in groups of 5, in 267 × 207 × 140 mm filter-topped polysulfone cages (370 cm^2^ floor area) (Type II-C, Tecniplast, Buguggiate, Italy) with corncob bedding (Scobis Due, Mucedola, SRL, Settimo Milanese, Italy), in an SPF animal facility. All animals were maintained under standard laboratory conditions: an artificial 12 h light/dark cycle (lights on from 8:00 to 20:00), with an ambient temperature of 21 ± 1 °C and a relative humidity of 50–60%. Mice were fed with a standard diet (4RF25 throughout the gestation and postnatal periods, and 4RF21 after weaning; Mucedola, SRL, Settimo Milanese, Italy) and water ad libitum. Health monitoring was performed according to the FELASA guidelines [55,56], confirming the Specified Pathogen Free status of sentinel animals maintained in the same room. Humane endpoints for the experiment were defined (i.e., 20% reduction in body weight, inability to reach food or water, presence of wounds on the body, dehydration), but not needed in practice, as the study period was conceived to include ages at which animals do not reach these endpoints.

### 2.4. Animal Treatment Groups

**Figure 1:**
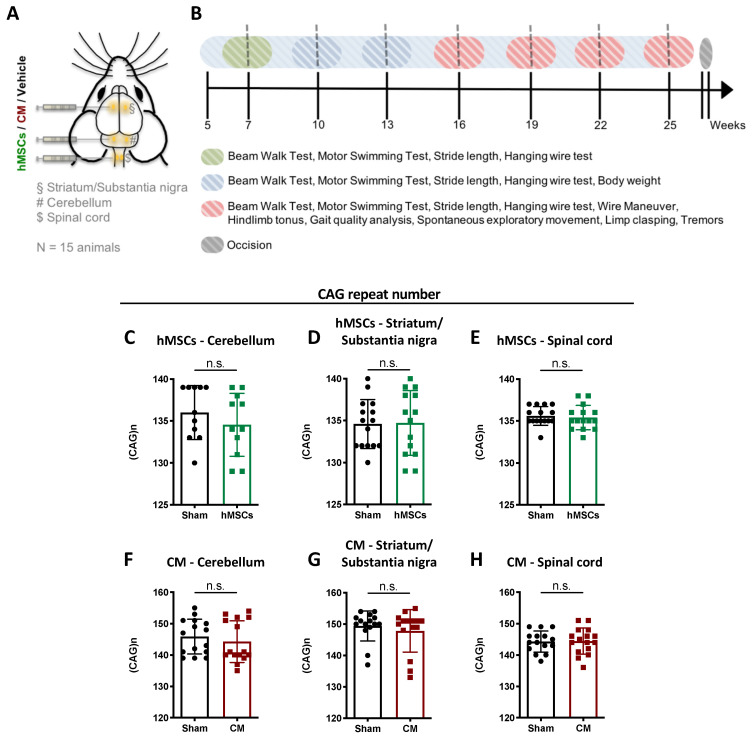
Experimental design: (**A**) Schematic representation of the experimental design. (**B**) Timeline for treatment and behavior tests. (**C**–**H**) No significant differences (n.s.) were observed in the mean CAG repeat size (mean ± SD) between control (sham) and treated groups (hMSCs: human mesenchymal stem cells; CM: culture media containing hMSC secretome) of transgenic animals within the same the preclinical trial.

At a pre-symptomatic age, five-week-old male mice were distributed among the different treatment groups in a randomized manner, and were submitted to stereotaxic surgery in different brain regions and the spinal cord (Figure 1A), and subjected to a behavioral testing design as shown in Figure 1B. The treatment initiation and the periodicity of the behavioral tests were chosen based on previous knowledge regarding disease onset and progression in this mouse model. A total of 156 male mice were used in these preclinical trials. The different groups of mice used in this study were as follows: wild-type sham (WT-sham, n = 14), CMVMJD135 transgenic animals (TG) sham (TG-Sham, n = 11 (as controls for TG-hMSCs)); TG-Sham, n = 15 (as controls for TG-CM), and administration of hMSCs or CM in the cerebellum (TG-hMSCs, n = 11; TG-CM, n = 15). For the interventions in the striatum/SN and spinal cord, only TG mice were used, and the following groups were analyzed: sham (Sham, n = 15 for striatum/SN and n = 15 for spinal cord), hMSC transplantation (hMSCs, n = 14 for striatum/SN and n = 15 for spinal cord), and CM administration (CM, n = 15 for striatum/SN and n = 16 for spinal cord). DNA extraction, animal genotyping, and CAG repeat size analyses were performed as previously described [7]. The mean CAG repeat size (Figure 1C–H) was not different among SCA3/MJD groups within the same intervention (mean ± SD; (min–max)_CAG_). hMSCs: cerebellum (Sham: 136 ± 3.22; (130–139)_CAG_; hMSCs: 135 ± 3.75; (129–139)_CAG_, Figure 1C); striatum/SN: (Sham: 135 ± 2.92; (130–140)_CAG_; hMSCs: 135 ± 3.85; (129–140)_CAG_, Figure 1D); spinal cord: (Sham: 136 ± 1.12; (133–137)_CAG_; hMSCs: 135 ± 1.45; (133–138)_CAG_, Figure 1E). CM: cerebellum (Sham: 146 ± 5.55; (139–155)_CAG_; CM: 144 ± 6.65; (135–154)_CAG_, Figure 1F); striatum/SN: (Sham: 149 ± 4.78; (137–154)_CAG_; CM: 148 ± 6.79; (133–155)_CAG_, Figure 1G); spinal cord: (Sham: 144 ± 3.39; (138–149)_CAG_; CM: 144 ± 4.15; (136–151)_CAG_, Figure 1H). Behavioral analyses were performed during the diurnal period, and the behavior tests were performed by the same experimenter, who was not blinded to the treatments. At the end of the preclinical trial, the animals were euthanized according to their final purpose: either by decapitation, or by exsanguination perfusion with saline or 4% paraformaldehyde (PFA; P6148-500G, Sigma-Aldrich, Darmstadt, Germany); in the latter case, the animals were deeply anesthetized with a mixture of ketamine hydrochloride (150 mg/kg; Imalgene, Merial, Lisbon, Portugal) and medetomidine (0.3 mg/kg; Dormitor, Cymedica, Horovice, Czech Republic).

### 2.5. Surgical Procedure

Five-week-old animals were anesthetized with ketamine–medetomidine (75 mg/kg; 1 mg/kg, intraperitoneally (i.p.)) diluted in 0.9% NaCl. Surgical procedures were performed under sterile conditions. For spinal cord interventions, the fur at the surgical site was shaved (1556-086, Moser, Unterkirnach, Germany), and the skin was disinfected with a mixture of 70% ethanol and chlorohexidine. For this procedure, the animals were placed in a prone position, and a dorsal midline incision was made at the cervical–thoracic level of the spine. The paravertebral muscles were retracted, the spinous processes and the laminar arc above the T1 vertebra (anatomical reference) were removed, and the spinal cord was exposed. Then, the animals were placed on a stereotaxic frame (51600, Stoelting, Wood Dale, IL, USA) to receive the hMSC transplants, CM injections (Hamilton Bonaduz AG., Bonaduz, Switzerland), and the respective vehicles at two different points of the cervical spinal cord. For the brain, the animals were placed on a stereotaxic frame, and one group was bilaterally injected with hMSC transplants or with the CM in the cerebellum (coordinates relative to the bregma: AP = −6.0 mm, ML = ±2.2 mm, DV = −3.6 mm [57]), and the other group in the substantia nigra (SN; coordinates relative to the bregma: AP = −3.2 mm, ML = ±1.1 mm, DV = −4.5 mm [57]) and striatum (coordinates relative to the bregma: AP = 0.62 mm; ML = ±2.2 mm, DV = −3.6 mm [57]). In the spinal cord, cerebellum, and SN, the animals received 1 μL of CM or 100,000 cells at each point/coordinate. In the striatum, because it is a much larger region of the brain, the animals received 2 μL of CM and 200,000 cells. The vehicle for the CM group was Neurobasal-A medium (10888-022, Thermo Fisher Scientific, Waltham, MA, USA), while for the transplanted group, alpha-MEM without serum was used, with the sham (control) animals injected with the same volumes as described above. The injection rate was 0.250 μL/min and, after each injection, the needle was left in place for 2 min to prevent backflow up the needle tract. Anesthesia was reversed using atipamezole hydrochloride (1 mg/mL; Antisedan^®^, Pfizer Inc., Brooklyn, NY, USA), and postoperative care was carried out using buprenorphine (0.05 mg/kg; s.c.; Butomidor^®^, Richter Pharma AG, Wels, Austria), administered twice a day, for a three-day period if needed.

### 2.6. Phenotype Assessment

#### 2.6.1. Body Weight 

Mice were weighed (EP2102CM, Ohaus, Switzerland) every 3 weeks, from 7 weeks of age until the end of the trial (25 weeks of age).

#### 2.6.2. Beam Walk Balance Test 

The test was performed as previously described [46]. Animals were trained for 3 days with the square beam (12 mm). At day 4, they were tested on the training beam and on two round beams (17 and 11 mm). If the animal fell or turned around on the beam, the trial was considered a failure. Each animal was given the opportunity to fail twice per beam. The time taken by the animal to traverse the beam was registered using a chronometer, and time was subtracted whenever the animal stopped on the beam. At later stages of disease, an additional 20 mm round beam was tested.

#### 2.6.3. Motor Swimming Test 

To analyze voluntary locomotion, mice were trained for 2 consecutive days (3 trials per animal) to traverse a clear Perspex water tank to a safe platform at the end. The Perspex tank was 100 cm long, and the platform was made of black Perspex. The latency to cross the water was measured from a distance of 60 cm. Water temperature was monitored and set to 23 °C using a thermostat [46].

#### 2.6.4. SHIRPA Protocol

In addition to the beam walk and motor swimming tests, a protocol for phenotypic assessment based on the primary screen of the SHIRPA protocol was performed, which resembles the diagnostic process of general neurological and psychiatric examination in humans [58]. A full description of the SHIRPA protocol is available at: https://www.mousephenotype.org/impress/ProcedureInfo?action=list&procID=1376 (accessed on 19 November 2021). Briefly, the tests were performed as follows:

- Spontaneous vertical exploratory movement—each mouse was placed in a viewing jar (15 cm diameter) for 5 min, and the number of vertical movements (rears) was registered;
- Spontaneous horizontal exploratory movement—mice were transferred to a 15-labeled-square arena (55 × 33 × 18 cm), and then a series of anatomical and behavioral features were registered. The number of squares travelled in the arena for 1 min was counted. Gait quality was scored by the same experimenter;
- Strength to grab—each animal was allowed to grab a metal grid (Series 012, Tecniplast, Buguggiate, Italy), and then was pulled backwards in the horizontal plane. The force applied by the animal was scored as active, mild, moderate, or absent;
- Hindlimb tonus—mice were properly restrained by the experimenter, and the hindlimb was smoothly pressed against the animal’s body. The resistance generated between the mouse’s hindlimb and the experimenter’s finger was scored as absent (no resistance), mild, moderate, or marked resistance;
- Limb clasping—each animal was picked up by the tail and slowly lowered towards a horizontal surface. The extension/contraction of the limbs were observed by the experimenter, and scored as absent or present in the back (one or both of the hind paws);
- Tremors—while the animals were in the viewing jar, the experimenter observed whether the animals presented tremors while completely immobile. Tremors were scored as absent, mild (discontinuous), or severe (continuous);
- Footprinting pattern—the footprinting test was used to evaluate the gait of the animals. To obtain footprints, the hind- and forepaws were covered with black or red nontoxic ink, respectively. For each run, a clean rectangular paper sheet was placed on the floor of the runway. Each animal was allowed to walk along a 100 × 4.2 × 10 cm corridor in the direction of an enclosed black box. An inclined corridor was used instead of a horizontal one, since mice have the tendency to run upwards to escape. Each animal was allowed to achieve one valid trial per timepoint. To score the severity of foot dragging, six consecutive steps were considered (0 = absent/mild, up to three steps; 1 = mild, more than three steps out of six; 2 = severe, all steps out of six). The stride length was measured manually as the distance between two pawprints. Three values were measured for six consecutive steps, and the mean value was derived (Figure S1);
- Hanging wire grip test—mice were placed on the top of a metallic grid (Series 012, Tecniplast, Buguggiate, Italy), which was inverted 180°, moving the animal towards the surface of the bench. The latency to fall from the grid was registered by the experimenter. The maximum time given for the test was 120 s;
- Wire maneuver test—mice were suspended by the tail and lowered onto a horizontal wire, and allowed to grab the wire using only the forelimbs. The latency to fall was scored, and a maximum time of 120 s was allowed.

### 2.7. Immunohistochemistry

Adult mice were subjected to the surgical procedure described above, in order to transplant hMSCs into the deep cerebellar nuclei of the cerebellum. Five different post-transplant timepoints were determined to confirm hMSC survival in the mouse brain: 48 h, 1 week, 4 weeks, 6 weeks, and 8 weeks post-transplantation of hMSCs. At each timepoint, mice were deeply anaesthetized and transcardially perfused with PBS, followed by 4% PFA in PBS. The brains were post-fixed overnight in fixative solution and embedded in paraffin (EG1140H, Leica, Germany); 4 μm thick paraffin sections were processed via immunohistochemistry for human nuclear antigen (HNA; MAB1281, 1:150, Merck Millipore, Lisbon, Portugal), and then developed with 3,3′-diaminobenzidine tetrahydrochloride (DAB) substrate (D5905, Sigma, Germany). Slice microphotographs (total 3–4 sections) were acquired using DPController software (Olympus, Spain) and a camera (Olympus DP70, Spain) attached to a motorized microscope (4E14135, Olympus BX61, Spain) using a 4 × 0.16 numerical aperture objective.

### 2.8. Statistics

The experimental unit used in this study was a single animal. Power analysis was used to determine the sample size, as previously described in [7]. The estimates of the required number of CMVMJD135 animals for specific behavioral tests and timepoints of analysis are described in [20]. Continuous variables with normal distributions (Shapiro–Wilk test *p* > 0.05) were analyzed using Student’s *t* test or mixed-design two-way ANOVA (factors were time and treatment; Tukey’s post hoc test was used for multiple comparisons). One-way ANOVA and repeated-measures ANOVA were used for comparisons between 3 groups. Univariate ANOVA was used to analyze the impact of body weight at each timepoint on motor performance tests (body weight was used as a covariate). All continuous data are shown as the as mean ± standard error of the mean (SEM). Behavioral data were subjected to the nonparametric Mann–Whitney U test or Kruskal–Wallis H test when variables were non-continuous, or when a continuous variable did not present a normal distribution (Shapiro–Wilk test *p* < 0.05). Outliers were considered at 1.5*IQR, and were excluded from the analyses. All statistical analysis was performed using SPSS 24.0 (IBM Corp., Armonk, NY, USA). A critical value for significance of *p* < 0.05 was used throughout the study.

## 3. Results

### 3.1. Single Administration of hMSC Secretome Mildly Improved the Motor Deficits of SCA3/MJD Mice

**Figure 2:**
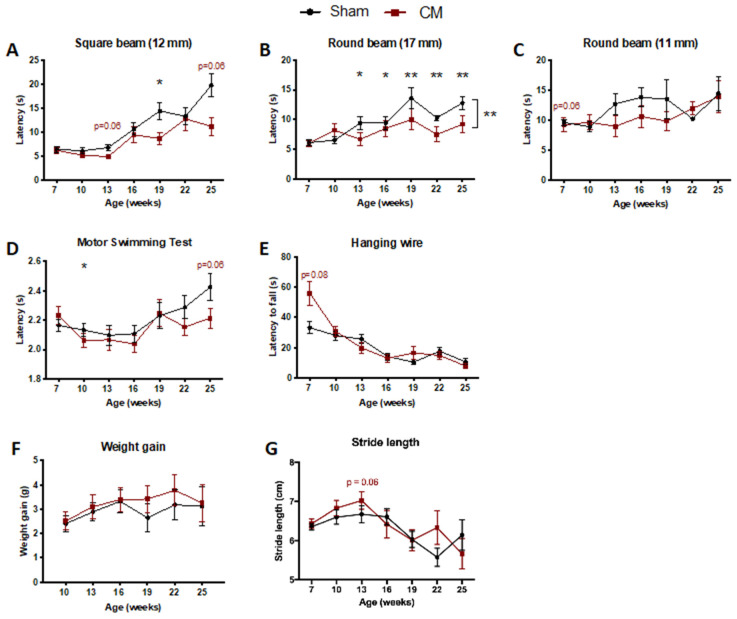
A single administration of CM to the cerebellum had a mild effect on the balance and motor deficits of SCA3/MJD mice. (**A**–**C**) Better performance of treated animals when traversing the less difficult beams in the beam walk test. Each bar corresponds to the mean of two consecutive trials in the (**A**) 12 mm square, (**B**) 17 mm round, and (**C**) 11 mm round beams. No improvement was observed in the motor coordination assessed by the (**D**) motor swimming test throughout disease progression, up to the end of the trial at 25 weeks of age. Treatment with CM in the cerebellum did not improve the muscle strength of SCA3/MJD animals as evaluated by the (**E**) hanging wire test. No differences were observed in (**F**) the body weight gain, or in gait quality as measured by the (**G**) stride length, for the CM-treated versus sham mice. Sham: control group; CM: human MSC secretome. For continuous variables with normal distribution, a mixed design two-way ANOVA was used for statistical analyses (**A**–**G**). Data is represented as the mean ± SEM. * *p* < 0.05; ** *p* <0.01.

We first investigated the potential of CM to promote amelioration of the motor decline and loss of muscular strength observed in a SCA3/MJD mouse model—the CMVMJD135 transgenic mouse, which shows a progressive SCA3/MJD-like phenotype that closely mimics human disease [7]. Because it has been suggested that the hMSC secretome per se may exert relevant neuroprotective actions in different neuropathological contexts [42,59,60], we tested the potential of CM to mitigate the neurological phenotype of the CMVMJD135 mice. For this, we administered CM (total protein concentration of 91 μg/mL) to the cerebellum (specifically into the deep cerebellar nuclei) of SCA3/MJD animals at an early age, when the symptoms could not yet be detected in these mice. Interestingly, CM treatment led to an improvement in the balance deficits of SCA3/MJD mice throughout disease progression (Figure 2A–C). Furthermore, a tendency towards an improvement was also observed in the swimming performance of these mice after a long period, as can be observed in Figure 2D. A similar trend was observed for muscular strength, where CM treatment appeared to improve the muscular strength of the SCA3/MJD limbs, as measured by the increased latency to fall from an inverted grid when compared to sham animals at 7 weeks of age. Nevertheless, the performance of the treated animals equaled that of the SCA3/MJD sham group at 10 weeks of age, suggesting that this effect on the muscle strength was not sustained over time (Figure 2E). No significant benefit of CM treatment was observed on body weight gain (Figure 2F), gait pattern and quality (Figure 2G and Figure S2A,B), exploratory spontaneous activity (Figure S2C,D), limb clasping (Figure S2E), or tremors (Figure S2F).

### 3.2. A Single Administration of hMSCs to the Cerebellum Led to No Overt Beneficial Effects on Balance, Motor Coordination, or Muscular Strength Loss of SCA3/MJD Mice

**Figure 3:**
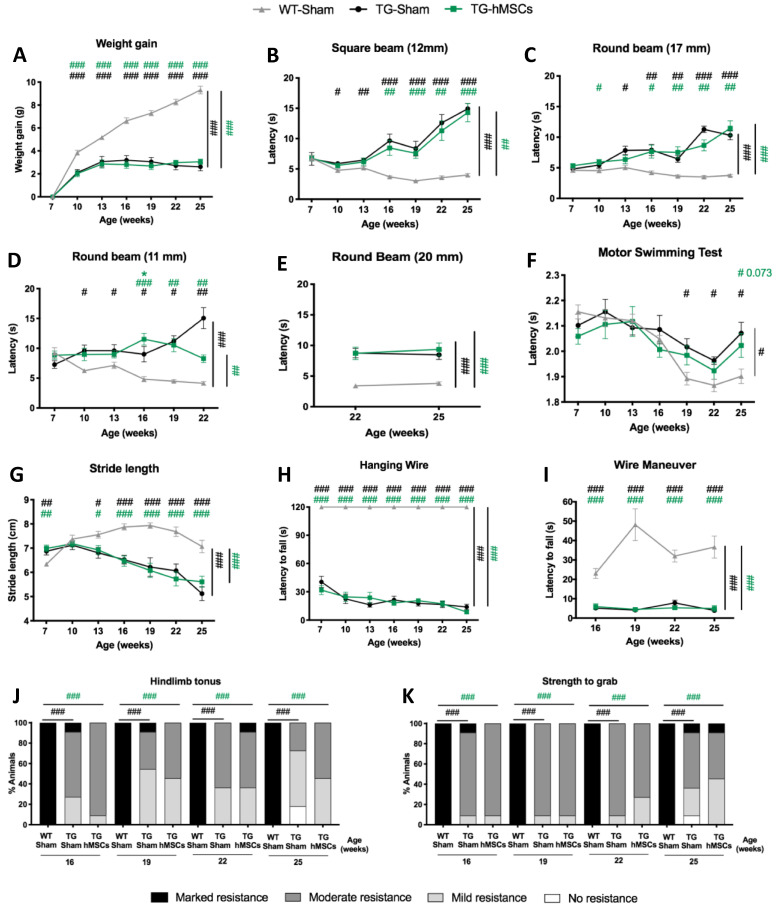
No overt effects on the balance, motor coordination deficits, or muscular strength of SCA3/MJD mice were promoted by a single administration of hMSCs to the cerebellum. (**A**) No impact on body weight gain was seen for the cerebellum-hMSC-transplanted group compared to the sham group. (**B**–**E**) No differences were observed between the performances of transgenic-treated animals (TG-hMSCs) and the sham group (TG-sham) in the beam walk test. The progression of phenotype was compared to sham wild-type littermates (WT-sham). Each bar corresponds to the mean of two consecutive trials using the (**B**) 12 mm square, (**C**) 17 mm round, (**D**) 11 mm round, and (**E**) 20 mm round beams. No impact was observed on motor coordination, assessed by the (**F**) motor swimming test, or on the (**G**) stride length of the treated mice throughout disease progression, up to the end of the trial at 25 weeks of age. Transplantation of hMSCs to the cerebellum did not improve the muscle strength of SCA3/MJD mice as determined by the (**H**) hanging wire test, (**I**) wire maneuver test, (**J**) hindlimb tonus, and (**K**) forelimb strength evaluated by the animal’s strength to grab a grid. WT-sham: wild-type littermates control group; TG-sham: transgenic littermates control group; TG-hMSCs: transgenic-treated animals with human MSCs. For continuous variables with normal distribution, a repeated-measures ANOVA was used for statistical analyses (**A**–**I**). Discrete variables were analyzed using a nonparametric Kruskal–Wallis H test (**J**,**K**). Data is represented as the mean ± SEM (**A**–**I**); in the case of (**J**,**K**) data is represented as frequencies. Asterisks represent differences between groups of transgenic animals * *p* < 0.05. Hash symbols represent differences between wild-type animals. # *p* < 0.05; ## *p* < 0.01; ### *p* < 0.001.

The therapeutic effect observed as a result of the administration of CM to the cerebellum of SCA3/MJD prompted us to evaluate the therapeutic effect of hMSC transplantation directly into this brain region. In addition to sham and treated SCA3/MJD mice, in this preclinical study, we added an extra control group—the sham WT-littermate animals—to allow us to determine the effect size of the treatment. Contrary to our hypothesis, hMSC transplantation had no beneficial effect on the motor deficits of SCA3/MJD mice. No alterations in body weight gain were detected between TG-sham and TG-hMSCs mice (Figure 3A). Additionally, transgenic hMSC-treated mice showed no improvement in their balance, based on their similar time taken to cross the different tested beams (both squared and round) compared to the TG-sham mice (Figure 3B–E) throughout the progression of the disease. Treatment with hMSCs also had no therapeutic effect on TG swimming performance (Figure 3F), nor on gait, as evaluated by measuring the animals’ stride length (Figure 3G). Muscular strength alterations were also measured, and hMSC treatment was not able to improve the deficits observed in sham animals (Figure 3H–K). Furthermore, no effects of hMSC treatment were observed on spontaneous exploratory activity (Figure S3A,B), gait quality (Figure S3C,D), limb clasping (Figure S3E), or tremors (Figure S3F).

### 3.3. hMSC Secretome Administration to the Basal Ganglia Improves the Motor Function of SCA3/MJD Mice

**Figure 4:**
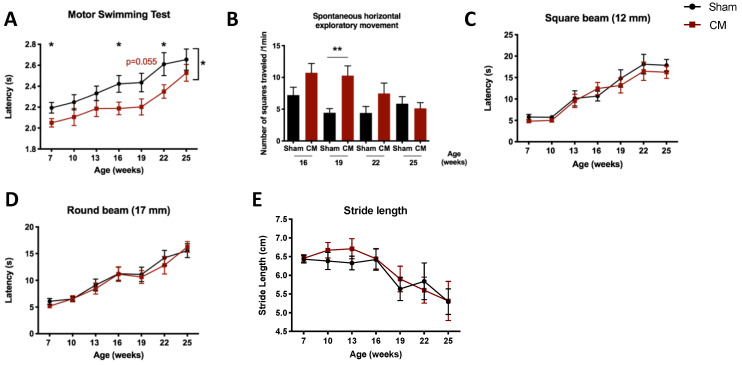
CM administration to the striatum/ substantia nigra improved the motor coordination of SCA3/MJD mice. (**A**) Better performance of treated animals in a motor coordination task assessed via the motor swimming test throughout disease progression, until the end of the trial at 25 weeks of age. (**B**) Transient improvement was observed in spontaneous exploratory movement in an open arena. (**C**,**D**) No effect was observed on the balance of SCA3/MJD mice, as assessed by the beam walk test. Each bar corresponds to the mean of two consecutive trials in the (**C**) 12 mm square and (**D**) 17 mm round beams. (**E**) No improvement was observed in the stride length of SCA3/MJD animals treated with CM in the striatum/ substantia nigra. Sham: control group; CM: human MSC secretome. For continuous variables with normal distribution, a mixed-design two-way ANOVA was used for statistical analyses (**A**,**C**–**E**). Discrete variables were analyzed using a nonparametric Mann–Whitney U test (**B**). Data is represented as the mean ± SEM of the different groups. * *p* < 0.05; ** *p* < 0.01.

Next, knowing the involvement of multiple brain regions in the pathogenesis of SCA3/MJD, we wanted to assess whether the transplantation of hMSCs or their secretome into other disease-relevant regions—namely, the midbrain (striatum and SN) and the spinal cord—would represent a potential therapy to counteract SCA3/MJD. CM treatment in the striatum/SN sustainably improved the motor coordination of treated SCA3/MJD mice (Figure 4A), and transiently improved their spontaneous exploratory movement (Figure 4B). No evident effects of this therapeutic approach were confirmed on balance (Figure 4C,D and Figure S4A,B), muscular loss (Figure S4C,D), gait pattern and quality (Figure 4E and Figure S4E,F), abnormal neurological reflexes (Figure S4G,H), or body weight gain (Figure S4I).

**Figure 5:**
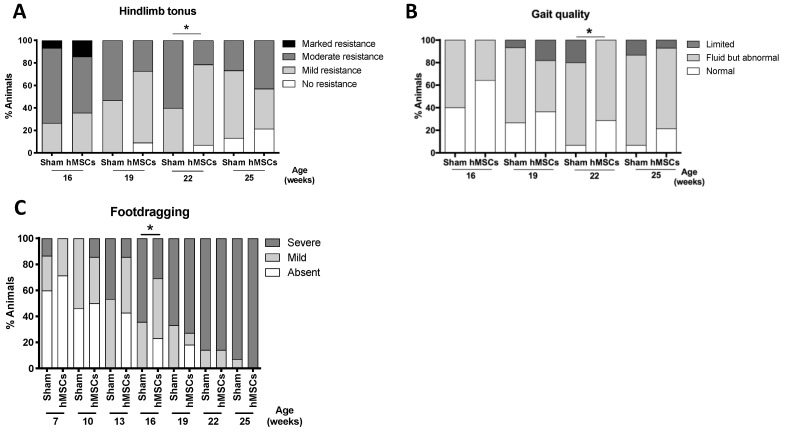
Transient effect of a single administration of hMSCs to the striatum/substantia nigra on the phenotype of SCA3/MJD mice. (**A**–**C**) Transient improvement in the muscle strength of SCA3/MJD animals when hMSCs were administered to the striatum/substantia nigra, as determined by (**A**) the hindlimb tonus test, and in gait quality, as evaluated (**B**) in an open arena and (**C**) by foot-dragging pattern analysis. Sham: control group; hMSCs: human MSCs. Categorical variables were analyzed using a nonparametric Mann–Whitney U test. Data is represented as frequencies. * *p* < 0.05.

In contrast, the injection of hMSCs in these brain regions showed no beneficial effects on SCA3/MJD mice (Figure S5), despite some transient effects on hindlimb tonus (Figure 5A), gait quality of animals when moving in an open arena (Figure 5B), and foot-dragging pattern (Figure 5C). 

**Figure 6:**
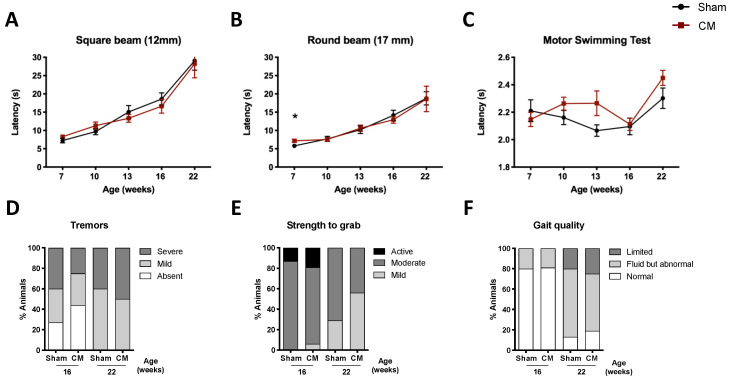
No overt therapeutic benefit was observed for CM administered to the spinal cords of SCA3/MJD mice. Poor performance of the treated animals was observed when traversing the less difficult beams in the beam walk test. Each bar corresponds to the mean of two consecutive trials in the (**A**) 12 mm square and (**B**) 17 mm round beams. No improvement was observed in motor coordination as assessed by the (**C**) motor swimming test throughout disease progression, up to the end of the trial at 22 weeks of age. No effect was observed on phenotypic parameters such as (**D**) tremors, (**E**) muscular strength—as measured by strength to grab a grid—and (**F**) gait quality. Sham: control group; CM: human MSC secretome. For continuous variables with normal distribution, a mixed-design two-way ANOVA (**A**–**C**) was used for statistical analyses. Categorical variables were analyzed using a nonparametric Mann–Whitney U test (**D**–**F**). Data is represented as the mean ± SEM (**A**–**C**); in the case of (**D**–**F**) data is represented as frequencies. * *p* < 0.05.

**Figure 7:**
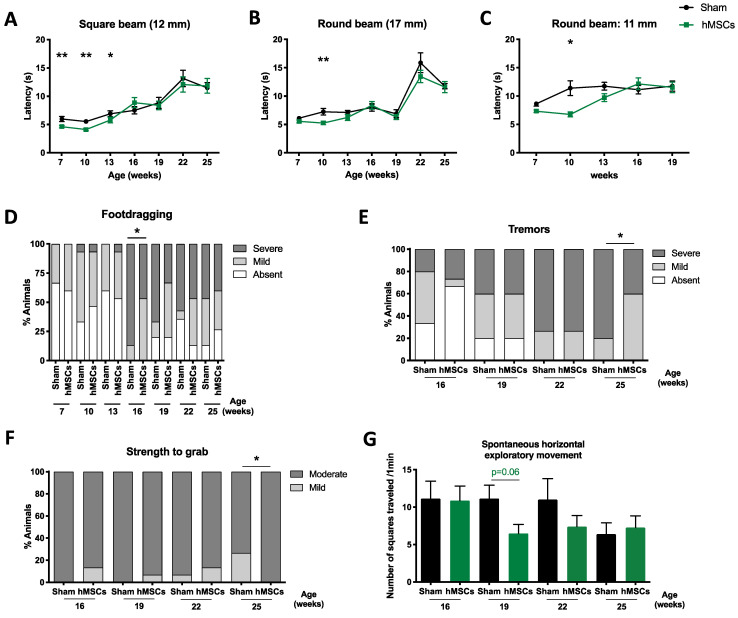
Limited effects of a single administration of hMSCs to the spinal cord on gait quality and neurological deficits in SCA3/MJD mice. (**A**–**C**) Administration of hMSCs to the spinal cord had a transient effect on the performance of treated animals when traversing the low-difficulty beams in the beam walk test. Each bar corresponds to the mean of two consecutive trials in the (**A**) 12 mm square, (**B**) 17 mm round, and (**C**) 11 mm round beams. Treatment also transiently reduced gait quality as assessed by (**D**) foot-dragging pattern, as well as the (**E**) tremors at late stages of the disease in SCA3/MJD animals. However, treatment also transiently worsened (**F**) muscle strength, as evaluated by the strength to grab a grid, and had no benefit on (**G**) the horizontal exploratory spontaneous activity of transgenic animals. Sham: control group; hMSCs: human MSCs. For continuous variables with normal distribution, a mixed-design two-way ANOVA (**A**–**C**) was used for statistical analyses. Categorical (**D**–**F**) and discrete (**G**) variables were analyzed using a nonparametric Mann–Whitney U test. Data is represented as the mean ± SEM (**A**–**C**); in the case of (**D**–**F**) data is represented as frequencies. * *p* < 0.05; ** *p* < 0.01.

Likewise, when CM or hMSCs were injected into the spinal cord, no overall benefit on the phenotype of SCA3/MJD mice was observed (Figure 6 and Figure S6; Figure 7 and Figure S7, respectively). Nevertheless, hMSC treatment transiently improved performance on the beam walk test (Figure 7A–C) and foot-dragging (Figure 7D), and led to a reduction in tremors at the later stages of the disease (Figure 7E).

In addition, for some parameters, spinal-cord-transplanted animals had a worse performance (Figure 7F,G and Figure S7). It is important to note that, unintendedly, animals treated with CM in the spinal cord had higher CAG repeat numbers (Figure 1H) when compared to the hMSC-transplanted group (Figure 1E); therefore (and given the known correlation between repeat length and disease severity in SCA3/MJD), these animals presented a more severe phenotype, making any potential beneficial effect more difficult to detect.

## 4. Discussion

In this work, we evaluated the therapeutic potential of hMSC transplantation or CM administration to different CNS regions known to be affected in SCA3/MJD: the cerebellum, the striatum/SN, and the spinal cord. Additionally, we also compared, for the first time, the effect of cell versus secretome treatments, with the idea that the use of hMSC byproducts could replace the hMSCs themselves, avoiding transplantation concerns and, thus, facilitating translation to clinical practice. We first compared the effects of presymptomatic hMSC transplantation to the different disease-relevant regions of the CNS. A single administration regimen was chosen, as this is more likely to be feasible in the clinical context.

We observed a more pronounced therapeutic benefit upon CM administration to the cerebellum when compared to the striatum/SN or to the spinal cord. Specifically, while CM treatment improved motor coordination deficits and increased performance in cerebellum-dependent motor tests—such as the beam walk test and the motor swimming test—and also improved gait quality and muscular strength in the cerebellum-treated group, the administration of CM to the striatum and SN led only to small and transient effects, mainly on swimming performance and exploratory activity. Additionally, administration of CM to the spinal cord had no evident beneficial effects. It should be noted that the worse performance observed in the balance and motor coordination of animals treated with CM to the spinal cord could be related to higher CAG repeats, which are known to be positively correlated with disease severity [61]. Interestingly, unlike the cerebellum and basal ganglia, the direct transplantation of hMSCs into the spinal cord led to a slight improvement in the balance of SCA3/MJD animals. This transient effect may be explained by the fact that the motor impairment of the transgenic mice—and, very likely, that of SCA3/MJD patients—is not solely due to dysfunction in one brain area, pointing to the need to consider broader transplantation approaches in future therapies. This may indeed be related to the fact that the best results were observed when treatment was applied to the cerebellum; in addition to receiving information from other brain regions [62], the cerebellum also projects to different important motor-related areas such as the vestibular nuclei and the deep cerebellar nuclei which, in turn, project to the upper motor neurons in the cortex, and to the spinal cord [62]. Therefore, treatment in the cerebellum could have an indirect impact on other brain regions that are also affected in disease, which receive inputs from the cerebellum and, thus, have a broader effect. Furthermore, our results also suggest that a single local treatment is not sufficient to produce the desired sustained efficacy, which is consistent with other studies, where it was shown that a repeated systemic administration of hMSCs led to more efficient and sustained improvements in the behavioral phenotype and neuropathology in different models of SCA3/MJD, compared with single intracranial administrations [38,39]. The same was observed in the case of SCA1; multiple intrathecal and intravenous injections of MSCs led to improvements in motor behavior and the morphology of the cerebellum for long periods after treatment [37,45]. Nevertheless, in our study design, we were still able to show some mild beneficial effects on balance deficits of the SCA3/MJD mice when we injected hMSCs into the spinal cord, 2–8 weeks after administration. It is important to highlight that this is a rather long preclinical trial (25 weeks) compared to others that have been performed (conducted for 8 [21,39] and 12 weeks [37,38]), which is very relevant in the context of a chronic disease. In fact, while for some clinical applications—such as spinal cord injury treatment—an acute intervention may be key to minimizing secondary injuries, in the case of SCA3/MJD and other neurodegenerative diseases that are progressive over a period of years, a chronic treatment is likely to be required. We then compared the effects of CM administration with those of hMSC transplantation, and found that when hMSCs were transplanted into the cerebellum, no motor improvements were observed. It is important to note that mice treated with CM in the cerebellum showed a higher CAG repeat length when compared to those transplanted with hMSCs, which we expected to lead to a higher disease severity, yet instead found better outcomes. In order to get more insight on hMSC survival post-transplantation, another set of mice were injected with hMSCs in the cerebellum, and cell survival was evaluated at different post-transplantation timepoints (Figure S8). From histological analysis using an HNA antibody, we were able to detect the survival of hMSCs after 48 h post-transplantation in the deep cerebellar nuclei (DCN) of the cerebellum (Figure S8D), compared to positive and negative controls (Figure S8A,B). Surprisingly, the HNA antibody seems to stain cells in the Purkinje layer for all of the timepoints analyzed (Figure S8C,E–H). In fact, previous studies have already reported the fusion of hMSCs with the Purkinje cell layer when injected in murine models of SCA1 [63] and EAE [64], suggesting migration of hMSCs to mediate neuroprotection or rescue of highly differentiated sites. Nevertheless, and in spite of these observations, according to our behavioral analysis, hMSCs injected into the cerebellum of SCA3/MJD mice do not cause an amelioration of disease symptoms. In contrast, even in animals with a higher disease severity, the CM treatment was able to improve the motor deficits of SCA3/MJD mice, making it a possible therapeutic strategy for SCA3/MJD. Additionally, this was the result of a single intracranial injection into one of the affected brain regions in the disease, therefore making it important to assess the effects of multiple CNS regions, as well as those of repeated systemic injections. It has been demonstrated—both in vivo and in vitro—that MSCs are able to secrete a broad repertoire of well-known neurotrophic factors—such as VEGF, GDNF, BDNF, IGF-1, and SCF—and important neuroregulatory factors such as 14-3-3 proteins, PEDF, galectin-1, cystatin C, clusterin, GDN, SEM74, and cadherin-2 [42,53,65,66], which promote neurogenesis, cell differentiation, angiogenesis, inhibition of apoptosis and glial scar formation, and neuronal and glial cell survival, thereby promoting neuroregeneration/neuroprotection and functional improvements in both acute and chronic models of disease [42,59,60,67]. In fact, it was previously described that treatment with human MSCs could enhance the levels of the neurotrophic factors IGF-1 and VEGF in cerebellar and serum samples of SCA3 animals [37]. Additionally, in an open-label clinical trial, it was demonstrated that subcutaneous IGF-1 treatment in SCA3/MJD patients could increase the SARA score after only 8 months; however, this benefit faded after 10–20 months [68]. Li et al. (2018) discussed whether the upregulation of the neurotrophin IGF-1 and the HSP70 chaperone molecular pathway could suppress the mutant ataxin-3 protein toxicity in MSC-treated SCA3 mice [38]. Moreover, MSCs can secrete BNDF, and valproic acid—a drug known to also increase the expression of this growth factor—has been shown to have beneficial effects in the treatment of SCA3/MJD [19].

Previous clinical trials in SCA patients reported the need for further detailed investigations of the molecular transformation of MSCs, in order to clarify the maximal passaging numbers that will ensure the long-term safety of hMSC therapy, and to elucidate the therapeutic mechanism [26,35,69]. This highlights the urgency of the need for readjustments in the protocols to improve treatment efficacy in the trials that are underway (http://www.clinicaltrials.gov, NCT01360164, NCT01489267, NCT01958177, NCT02540655, NCT03378414; accessed on 25 July 2021), as well as for the harmonization of the protocols used and, possibly, the formulation of guidelines for these trials, favoring higher sample sizes and reducing the risk of potential bias [69]. From the lessons we have learned from the completed clinical trials, and because this and other preclinical studies suggest that a multiple MSC transplantation regimen could be a promising therapeutic strategy for the treatment of SCA3/MJD, it is imperative to address some concerns, such as (1) the possible risks of repeated (and systemic) transplantation, (2) defining the least invasive and effective route of administration, (3) determining which types of cells offer the best potential to treat this particular disease, (4) anticipating patients’ responses to treatments according to disease course, (5) defining the predicted actions of stem cells and the outcomes to be expected, and (6) establishing the therapeutic value of hMSC transplantation at a post-symptomatic stage.

## 5. Conclusions

In this work, we showed that single administration of hMSCs and their secretome to individual disease-relevant regions of the CNS has minor therapeutic effects on different aspects of the phenotype of SCA3/MJD mice. Despite this mild and, in some cases, transient therapeutic effect, the results obtained in the present study position these advanced therapies as promising candidates to halt the progression of SCA3/MJD, requiring further development. In the future, these cellular or cell-derived therapies could be included as part of multitarget therapeutics for SCA3/MJD.

## Data Availability

Not applicable.

## Supplementary Materials

The following are available online at https://www.mdpi.com/article/10.3390/biomedicines9121754/s1: Figure S1: Footprint analysis; Figure S2: CM cerebellum; Figure S3: hMSCs cerebellum; Figure S4: CM striatum/SN; Figure S5: hMSCs striatum/SN; Figure S6: CM spinal cord; Figure S7: hMSCs spinal cord; Figure S8: Survival of hMSCs in the murine brain.
