# Supplementary material for: Preclinical Assessment of Mesenchymal-Stem-Cell-Based Therapies in Spinocerebellar Ataxia Type 3
Source: Biomedicines. 2021 Nov 24;9(12):1754. doi: 10.3390/biomedicines9121754 (PMC8698556; doi:10.3390/biomedicines9121754)
Supplement: Supplementary file 1 [file biomedicines-09-01754-s001.zip › biomedicines-1444271-supplementary.pdf]

Supplementary Materials

Figure S1 – Representative picture of the Footprint analysis

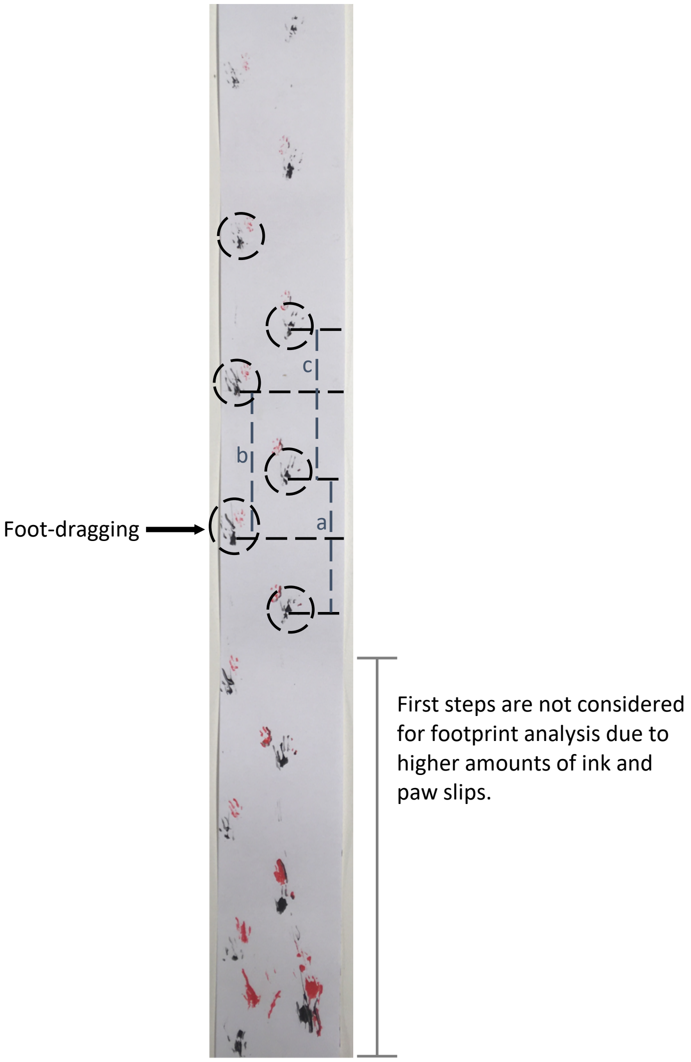

Figure S1 – Representative paper stripe of the footprint analysis of SCA3/MJD mice. A representation of six consecutive paws used for the analysis is indicated by circle lines. (**a, b, c**) represent the there stride measures. The arrow in the left side of the stripe, indicates foot dragging. The foot dragging pattern was classified considering the six consecutive steps (0 = absent/mild, up to three steps; 1 = mild, more than three steps out of six; 2 = severe, all steps out of six). The first steps were not considered for the footprint analysis because higher amounts of ink cause the paws to slip. The apparatus used for this test was a runway inclined corridor with 100 x 4.2 x 10 cm.

Figure S2 - CM Cerebellum

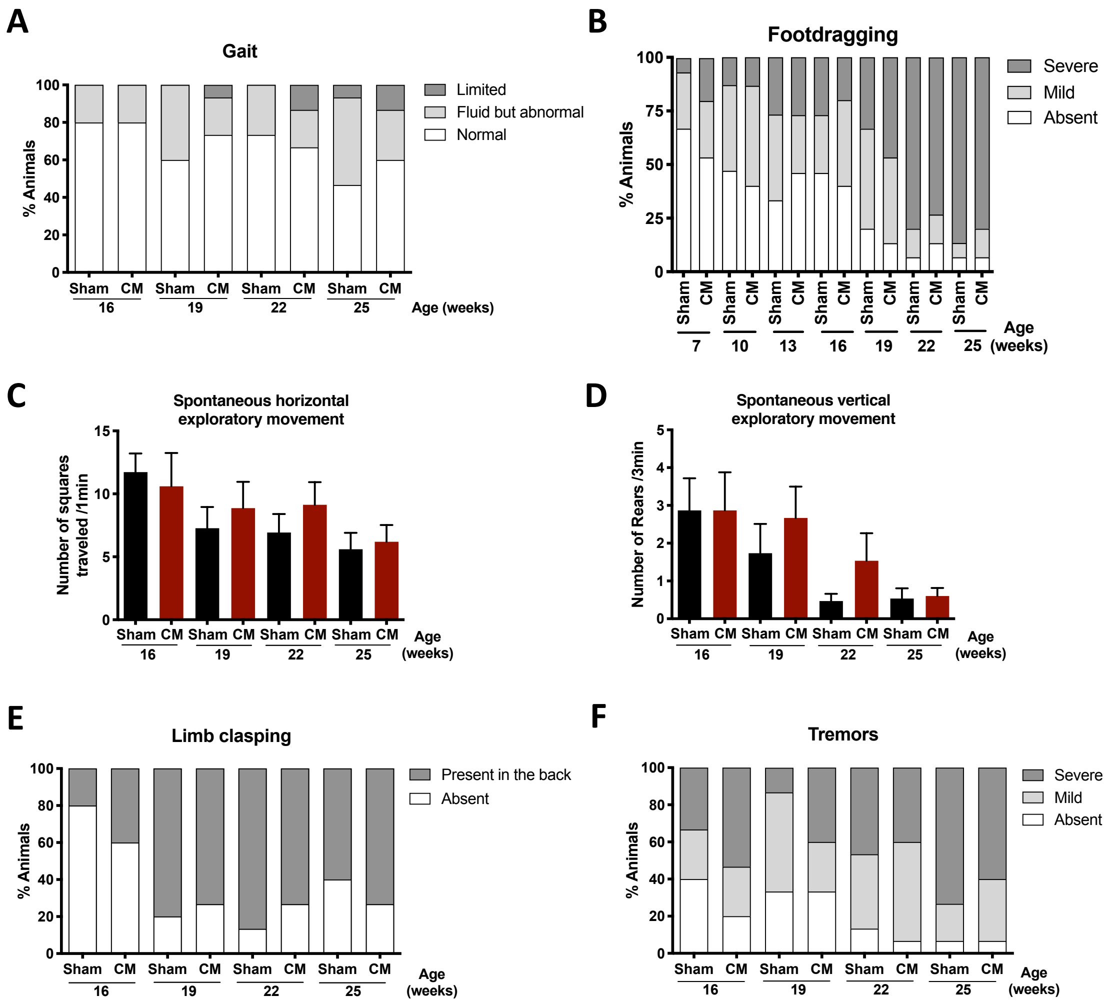

Figure S2 – Phenotypic parameters in which there was no effect of CM administration into the cerebellum of SCA3/MJD mice. CM administration into the cerebellum did not improve gait quality as evaluated **(A)** in an open arena and **(B)** by footdragging pattern analysis, and had no effect in the **(C)** horizontal and **(D)** vertical exploratory spontaneous activity, nor in **(E)** limb clasping and in **(F)** tremors of SCA3/MJD mice. Sham: control group; CM: human MSC secretome. Categorical and discrete variables were analyzed by nonparametric Mann-Whitney U test (A-F). Data is represented as frequencies (A,B,E,F); in the case of (C,D) data is represented as mean ± SEM.

Figure S3 - hMSCs Cerebellum

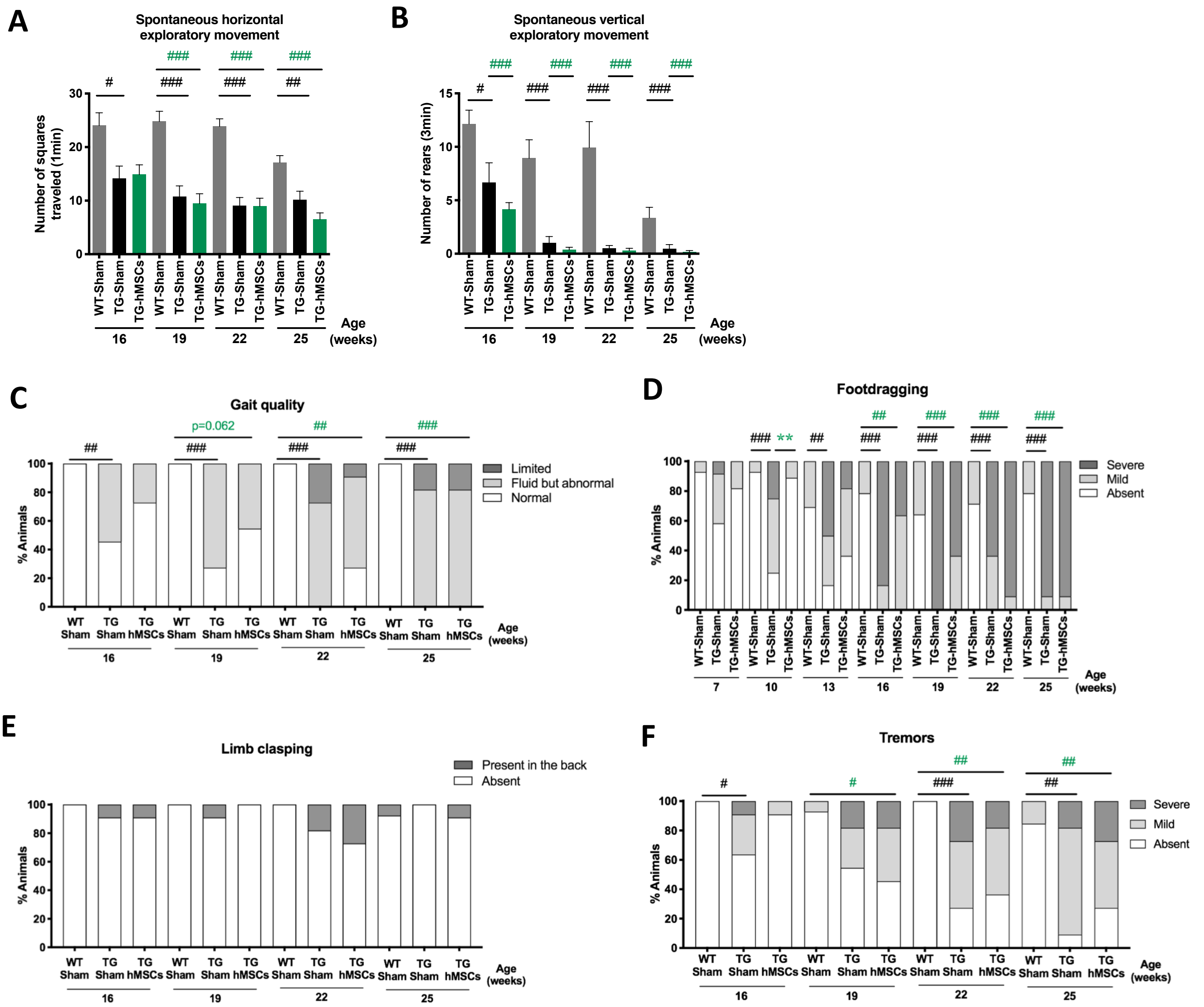

Figure S3 – Phenotypic parameters in which there was no effect of hMSC transplantation into the cerebellum of SCA3/MJD mice. (**A**) The horizontal and (**B**) vertical exploratory spontaneous activity were not improved across disease progression. No benefit observed on (**C**) gait quality as evaluated in an open arena nor on (**D**) footdragging pattern analysis. No effect of hMSC transplantation into the cerebellum in the (**E**) limb clasping and in (**F**) tremors of SCA3/MJD mice. WT-Sham: wild-type littermates control group; TG-Sham: transgenic littermates control group; TG-hMSCs: transgenic treated animals with human MSCs. Discrete (A, B) and categorical (C-F) variables were analyzed by nonparametric Kruskal-Wallis H test. Data is represented as the mean ± SEM (A-B); in the case of (C-F) data is represented as frequencies. Asterisks represent differences between groups of transgenic animals. ** p<0,01. Hash symbols represent statistical differences to wild-type animals. # p<0,05; ## p<0,01; ### p<0,001.

Figure S4 - CM Striatum/ SN

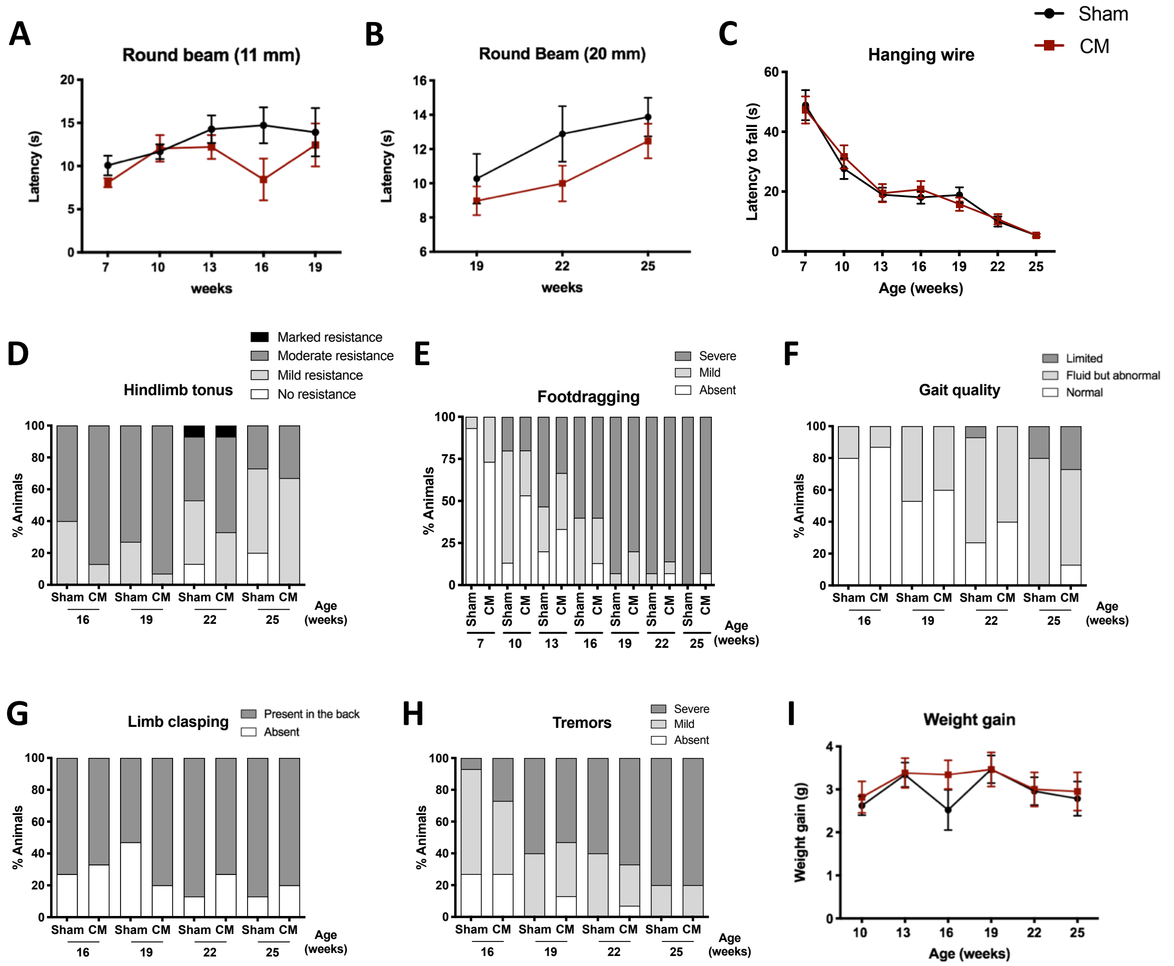

Figure S4 – Phenotypic parameters in which there was no effect of CM administration into the striatum/ substantia nigra of SCA3/MJD mice. No differences in **(A-B)** the beam walk test for the CM versus Sham groups. Each bar corresponds to the mean of two consecutive trials in the **(A)** 11 mm and **(B)** 20 mm round beams. No significant differences were seen muscular strength in the **(C)** hanging wire test and in the **(E)** hindlimb tonus. No effect was observed on **(F)** foot-dragging or gait quality **(E)**, neither on the neurological parameters such as **(G)** limb clasping and **(H)** tremors. No differences in the **(I)** body weight gain for CM-treated animals when compared to sham group. Sham: control group; CM: human MSC secretome. For continuous variables with normal distribution a mixed design 2-way ANOVA (A-C, I) was used for statistical analyzes. Categorical variables were analyzed by nonparametric Mann-Whitney U test (D-H). Data is represented as mean ± SEM (A-C, I); in the case of (D-H) data is represented as frequencies.

Figure S5 - hMSCs Striatum/ SN

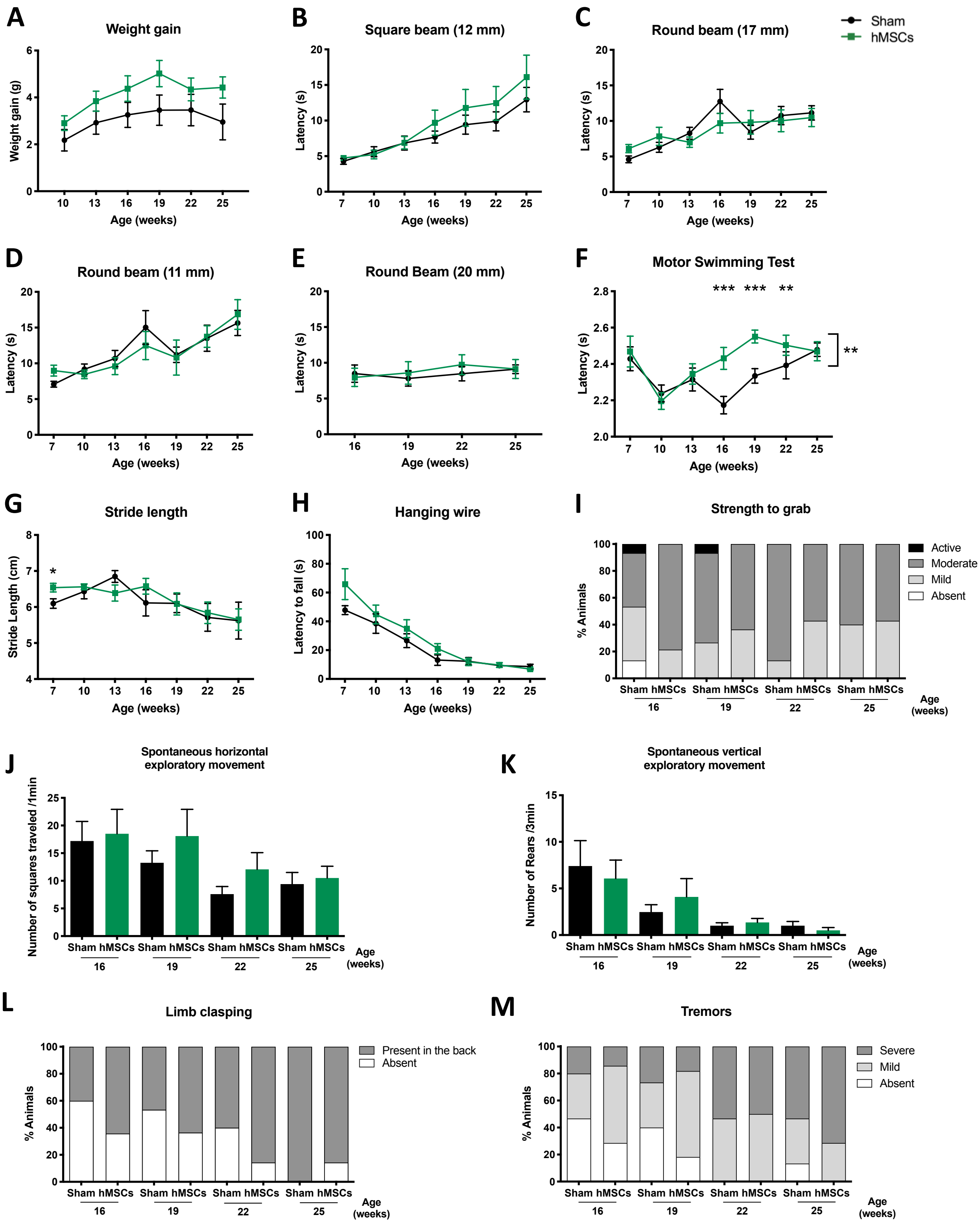

Figure S5 – Phenotypic parameters in which there was no effect of transplantation of hMSCs into the striatum/substantia nigra of SCA3/MJD mice. No significant differences were seen in the **(A)** body weight gain and **(B-E)** the beam walk test for the hMSC-transplanted versus Sham groups. Each bar corresponds to the mean of two consecutive trials in the **(B)** 12 mm square, **(C)** 17 mm round, **(D)** 11 mm and **(E)** 20 mm round beams. hMSC administration worsened the motor coordination of SCA3/MJD animals as assessed by **(F)** motor swimming test. No effect on muscle strength was observed as determined by the **(G)** stride length, **(H)** hanging wire tests and **(I)** strength to grab a grid, on **(J)** horizontal and **(K)** vertical exploratory spontaneous activity, on **(L)** limb clasping, and on **(M)** tremors. Sham: control group; hMSCs: human MSCs. For continuous variables with normal distribution a mixed design 2-way ANOVA (A-H) was used for statistical analyzes. Discrete (J, K) and categorical (I, L) variables were analyzed by nonparametric Mann-Whitney U test. Data is represented as mean ± SEM (A-H, J, K); in the case of (I, L, M) data is represented as frequencies. * $p<0,05$; ** $p<0,01$; ***$p<0,001$.

Figure S6 - CM Spinal cord

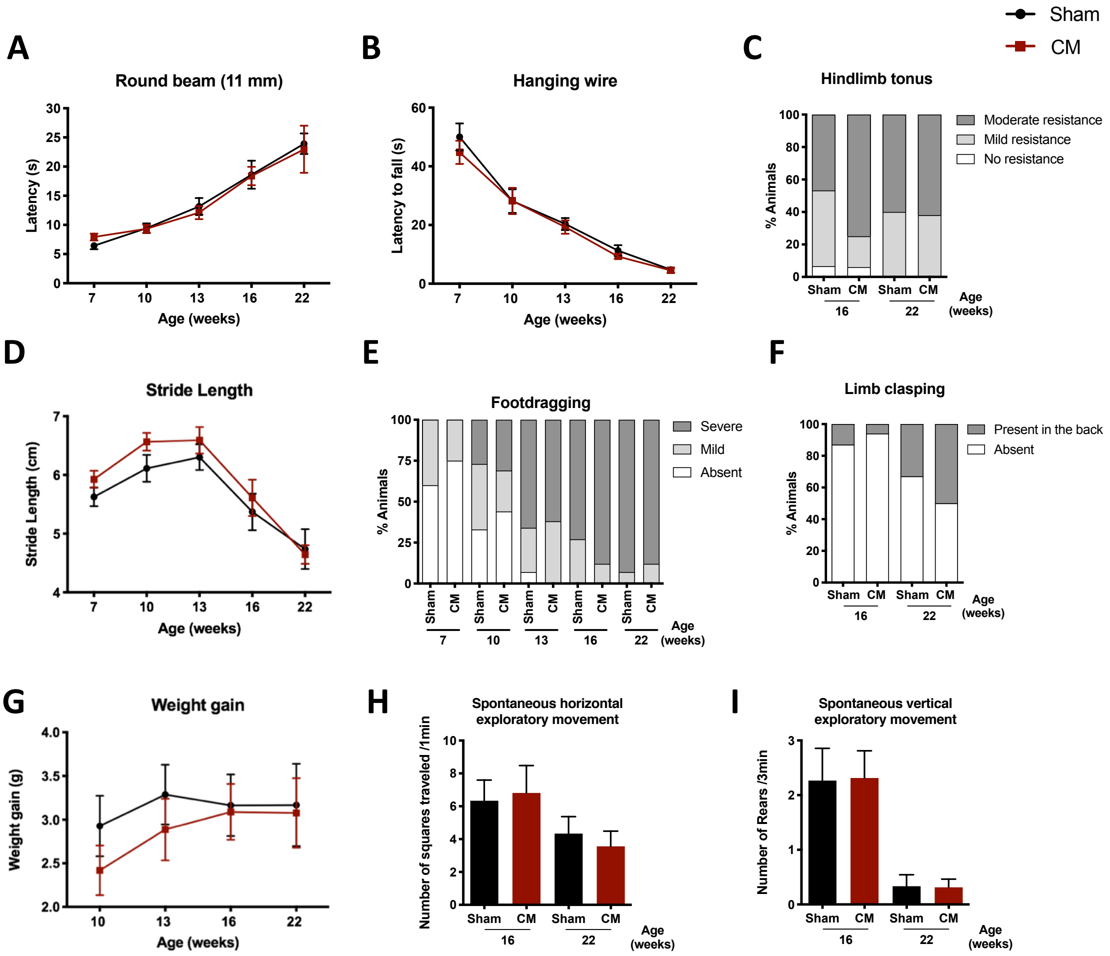

Figure S6 – Phenotypic parameters in which there was no effect of a single administration of CM into the spinal cord of SCA3/MJD mice. No significant differences in the ability to cross the most difficult beam, **(A)** 11 mm round beam, in loss of muscular strength **(B, C)** and on gait quality **(D, E)**. No significant differences were observed in the **(F)** limb clasping neurological phenotype nor on the **(G)** body weight gain and **(H-I)** spontaneous exploratory activity of SCA3/MJD mice. Sham: control group; CM: human MSC secretome. For continuous variables with normal distribution a mixed design 2-way ANOVA (A, B, D, G) was used for statistical analyzes. Discrete (H, I) and categorical variables (C, E, F) were analyzed by nonparametric Mann-Whitney U test. Data is represented as mean ± SEM (A, B, D, G-I); in the case of (C, E, F) data is represented as frequencies.

Figure S7 - hMSCs Spinal Cord

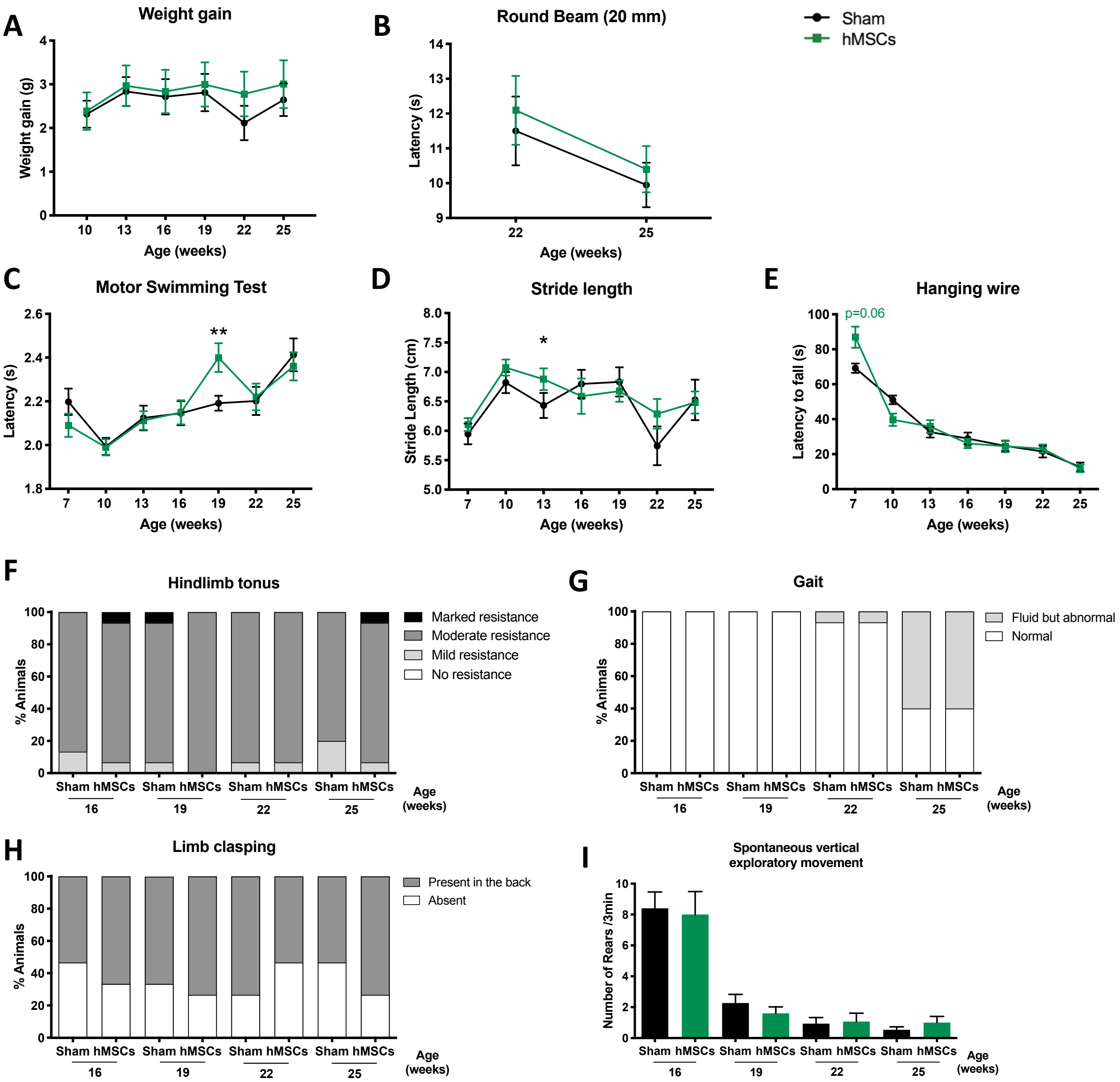

Figure S7 – Phenotypic parameters in which there was no effect of a single injection of hMSCs transplant into the spinal cord of SCA3/MJD mice. No significant differences in the **(A)** body weight gain, **(B)** on the ability to cross a 20 mm round beam, in motor coordination as assessed by **(C)** the motor swimming test, and on the muscle strength of SCA3/MJD animals as assessed by the **(D)** stride length, **(E)** hanging wire and **(F)** hindlimb tonus, nor on gait quality as evaluated in **(G)** an open arena, or on neurologic parameters as **(H)** limb clasping and **(I)** no effect on spontaneous exploratory vertical activity of transgenic animals. Sham: control group; hMSCs: human MSCs. For continuous variables with normal distribution a mixed design 2-way ANOVA (A-E) was used for statistical analyzes. Categorical (F-H) and discrete (I) variables were analyzed by nonparametric Mann-Whitney U test. Data is represented as mean ± SEM (A-E, I); in the case of (F-H) data is represented as frequencies. * $p<0,05$; ** $p<0,01$.

Figure S8 – Survival of hMSCs in the mouse brain

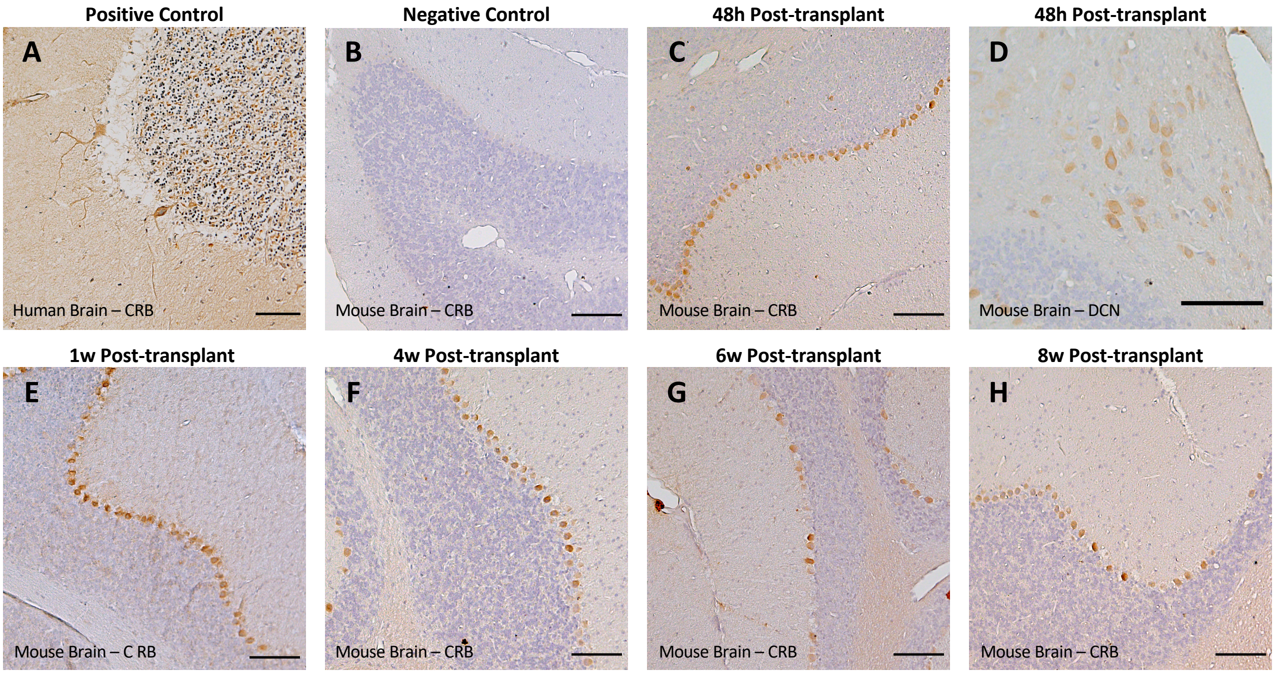

Figure S8 – Histological analysis of the survival of hMSC in the cerebellum of SCA3/MJD mice. Staining using DAB-reaction represent human nuclear antigen (HNA). Upper panel: **(A)** as positive control a sample of human cerebellum (CRB) was used; **(B)** a sample of mouse CRB was used as negative control; **(C)** at 48 hours post-transplant we can observe staining in the Purkinje layer of the mouse CRB and **(D)** at the site of injection, in the deep cerebellar nuclei (DCN) an inset picture in shown. Bottom panel: **(E-H)** Images of mouse CRB for additional timepoints post-transplant; (E) 1 week, (F) 4 weeks, (G) 6 weeks and (H) 8 weeks post-transplant. For all images, N = 2. Scale bars = 100 μm; 10x optical magnification.
